# The Percentage System Amongst Dentists

**Published:** 1851-01

**Authors:** 


### ARTICLE V.

### *The Percentage System amongst Dentists.*

Some few years since there appeared in one of our leading periodical publications, a very astounding and thorough exposure of the systematic frauds that prevailed among nearly all classes of tradesmen employed by the gentry and aristocracy, by the allowance to domestics, servants, of a percentage or a discount upon the amount of their master's bills. The practice was not confined to the purveyors of meat, bread, groceries, and "long sixes," but was found to prevail very extensively among the more influential and apparently respectable establishments of the West End. "Black-Mail was regularly demanded and exacted, and as a matter of course charged upon the customer's bills. The exposure, however, produced the most satisfactory results ; the wholesale system of knavery was unmasked ; masters and mistresses began to look after their own interests, and paid their own bills instead of entrusting their purses to the less scrupulous servant or the tender mercies of your genteel fraudulent tradesman. Could we only hope that the curious revelations we are about to make would produce an equally satisfactory and necessary reformation in another part of our social system we should deem ourselves amply repaid.

It will scarcely be credited that so mean and dishonorable a practice would be found to exist in any but the lowest and most unprincipled class of tradesmen. It will naturally be supposed that in moral and intellectual England, whose commercial integrity is recognised throughout every quarter of the globe, these sort of frauds were perpetrated merely by scheme-ing tradesmen and mercenary valets, and that the professors of medicine and surgery would stand not only pure, but above even the shadow of suspicion. Few will believe, without satisfactory evidence, that men of education and principle, would become parties to so gross a violation of principles, and imposition upon the public confidence, reposed in professional men, whatever may have been the case at the period when these domestic frauds were first made public we cannot say ; but we can assert, that of late, some very extraordinary disclosures have been made, calculated to excite just as great a sensation among the profession, as the former ones did in the regions of flunkeyism, with this unfortunate difference, that in the one case they were perpetrated by men whose character and station was little compromised by the exposure, while in the other they must tend materially to degrade a highly honorable class of men, and to shake public confidence in professional integrity, and honor. In support of our observations we submit the following very curious circular note, sent by a dentist, to a medical man, residing in the country.

George street, Hanover square, London.

Sir :—I hope you will excuse my troubling you with this communication. I am a dentist of considerable practice, but wish to extend it by the recommendation of medical men. If you will do me the favor to recommend those of your patients who require a dentist to my care, I shall be most happy to present you with a *liberal compliment* upon every case you send me.

I am, &c.

——— ———

That there are men in every calling, including dental surgery, who will stick at nothing to obtain their ends, we are fully aware. And whatever the profession generally may think, we are not very much surprised at the discovery of this issue of dental scrip, by speculative practitioners, which entitles parties bringing pigeons to be plucked. We have reason further to know that the speculation has been systematically carried out to a considerable extent, and that the department in which it flourishes with the greatest vigor, is that of dentistry. It is a well known fact, that we have grafted upon the dental profession, in this country, many rotten branches who have no identity with the parent stock. Men of little practice and less principle, who, while following apparently a useful and respectable calling, possess a very limited amount of either education or character, and endeavor to compensate for their deficiencies in both by resorting to all sorts of chicanery and cunning, which they frequently bring to bear upon the public, with the most profitable results.

In every bubble company, and in every speculation, nothing is more common than to find some psuedo foreign count, or baron, who condescendingly allows his name, from purely philanthropic motives to be put forward as a director or secretary; but what are the disinterested exertions of such men to those of the dental practitioner, who having already a "considerable practice," which one would suppose required his best attention, obligingly sacrifices it from a wish to extend it, among that class of her majesty's lieges who have not yet had the benefit of his professional services; the question presents some of these extraordinary contradictions and anomalies that cannot fail to be highly instructive to any reflective mind, while the ingenuity with which the disgraceful compact is suggested, by means of the tempting bait, held out to the "decoy duck," evinces a degree of assurance and of talent, that might claim our admiration, if employed in a more worthy cause. But the specimen of "shingles to catch woodcocks," which we have quoted above, is not the only illustration we can furnish—the examples are neither isolated nor rare as the following advertisement from the "Lancet" will show.

*To Medical Men and Chemists.*—The advertiser being a *Surgeon Dentist*, attached to a public institution, and in private practice will engage to allow twenty-five per cent. for all patients sent to him for professional advice. Address A. B., Post Office, Leigh street, Burton Cresent, London.

We quote another from the "Times," as follows:

*To Medical Men.*—A dentist in full practice at the West End, will remunerate handsomely any medical gentleman who will send him patients. Address under cover to Y. Z. W. Balares, Regent street, London.

This last *rara avis* is perhaps the worst sample of all, Here is an unconscionable Shylock for you—who being, according to his own statement, "in full practice," opens his insatiable maw to swallow up the few stray cases that the poor struggling and deserving men, close at hand, are starving for. But, in sober seriousness, it is much to be regretted that there should be educated men, members of a liberal and philanthropic profession, in which the noblest feelings of our nature are being constantly called into action, who would lend themselves to such fraud.

When the judgment and advice of a medical man are sought under these circumstances, the utmost confidence and security are placed in his integrity; and the patient asks it in the belief that his medical adviser is entirely exempt from all personal considerations in the matter. He presumes that in relying upon his recommendation, he may rest assured of the judgment and skill of the dental operator, of his ability to perform his duty, and that the charges will not be extravagant or unreasonable. What then must be said of the professional man who will degrade himself by either sharing in or countenancing these extortions, who so far forgets the dignity of his calling, and his moral obligations to his patient, as to pander to one of the worst vices of his calling, which is based upon quackery and incompetence, and which involves a breach of professional confidence?

That there are medical men who lend themselves to these practices, and hire out their professional character for a percentage, there can be no doubt; and that there are persons calling themselves respectable dental practitioners or extortioners who know that such men can be procured, it is equally reasonable to infer, as the circular and advertisements we have quoted will testify: and thus we find the representations of two learned and respectable professions, hanging together to levy and share black-mail on the unfortunate patient in a similar manner to the retailers of short sixes and long fours, and with the same amount of gentlemanly feeling and consideration as the lackeys of a gentleman's establishment.

Although the percentage system is carried on, *sub rosa*, to a considerable extent, sooner or later, exposure will follow; the misdeeds of these men will be made public, and the abettors held to public indignation and contempt. It cannot be too often impressed upon all candidates for professional favors that, in the long run, honesty will be found the best policy; and that a reputation based upon such a disreputable system is as evanescent and as perishable as the unwholesome breath that has created it—*spectemur agendo*, say we. R.

